# Two Cholesterol Recognition Amino Acid Consensus Motifs of GP64 with Uncleaved Signal Peptide Are Required for Bombyx mori Nucleopolyhedrovirus Infection

**DOI:** 10.1128/Spectrum.01725-21

**Published:** 2021-12-22

**Authors:** Bifang Hao, Wenbin Nan, Ying Xu, Lin Liu, Na Liu, Jinshan Huang

**Affiliations:** a Jiangsu Key Laboratory of Sericultural Biology and Biotechnology, School of Biotechnology, Jiangsu University of Science and Technology, Zhenjiang, Jiangsu, People’s Republic of China; b Key Laboratory of Genetic Improvement of Sericulture in the Ministry of Agriculture, Sericultural Research Institute, Chinese Academy of Agricultural Science, Zhenjiang, Jiangsu, People’s Republic of China; Oklahoma State University, College of Veterinary Medicine

**Keywords:** BmNPV, *Bombyx mori*, GP64, baculovirus, cholesterol recognition amino acid consensus, membrane fusion, signal peptide

## Abstract

The signal peptide (SP) of integrated membrane proteins is removed cotranslationally or posttranslationally in the endoplasmic reticulum, while GP64, a membrane fusion protein of Bombyx mori nucleopolyhedrovirus (BmNPV), retains its SP in the mature protein and virion. In this study, we revealed that uncleaved SP is a key determinant with additional functions in infection. First, uncleaved SP endows BmNPV with strong virulence; second, SP retention-induced BmNPV infection depends on cholesterol recognition amino acid consensus domain 1 (CRAC1) and CRAC2. In contrast, the recombinant virus with SP-cleaved GP64 has reduced infectivity, and only CRAC2 is required for BmNPV infection. Furthermore, we showed that cholesterol in the plasma membrane is an important fusion receptor that interacts with CRAC2 of GP64. Our study suggested that BmNPV GP64 is a key cholesterol-binding protein and uncleaved SP determines GP64's unique dependence on the CRAC domains.

**IMPORTANCE** BmNPV is a severe pathogen that mainly infects silkworms. GP64 is the key membrane fusion protein that mediates BmNPV infection, and some studies have indicated that cholesterol and lipids are involved in BmNPV infection. A remarkable difference from other membrane fusion proteins is that BmNPV GP64 retains its SP in the mature protein, but the cause is still unclear. In this study, we investigated the reason why BmNPV retains this SP, and its effects on protein targeting, virulence, and CRAC dependence were revealed by comparison of recombinant viruses harboring SP-cleaved or uncleaved GP64. Our study provides a basis for understanding the dependence of BmNPV infection on cholesterol/lipids and host specificity.

## OBSERVATION

**FIG 1:**
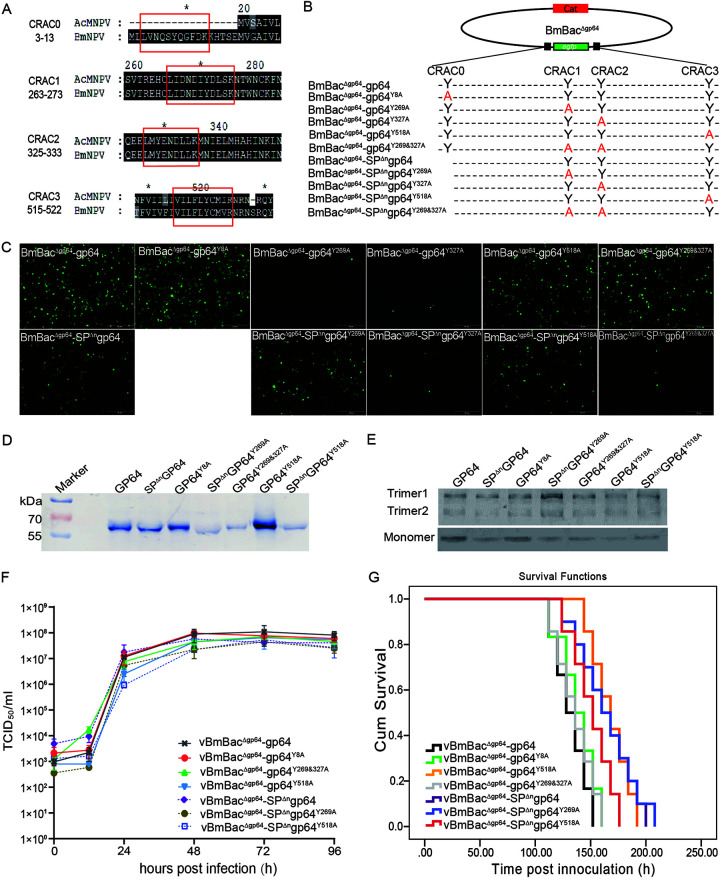
Recombinant viruses expressing wild-type GP64 and SP-cleaved SP^Δn^GP64 with alanine substitution mutations in CRAC domains. (A) Comparison of CRAC domains of GP64 in AcMNPV and BmNPV. CRAC motifs are boxed in red. (B) Schematic diagrams of CRAC mutant bacmids. CRAC mutants were constructed by overlapping PCR. Key tyrosine residues were replaced with alanine (red letters), and then these mutants and the control were reintroduced into the *gp64*-null bacmid by Tn7-based transposition into the *ph* locus. (C) Analysis of virus replication in BmN cells. The recombinant bacmids were transfected into BmN cells with H4000 transfection reagent according to the protocol. Fluorescence microscopy images were recorded at 96 h p.t. (D) Detection of CRAC-mutated GP64 in BVs. BVs were collected by ultracentrifugation and subjected to SDS-PAGE separation and Western blotting with a GP64 antibody. (E) Western blotting of the expression and trimerization of wild-type and CRAC-mutated GP64 proteins on nonreducing (upper) and reducing (lower) gels. (F) One-step growth curve analysis of viruses expressing CRAC mutant constructs. BmN cells were infected with viruses expressing the wild-type protein or CRAC mutant at a multiplicity of infection (MOI) of 5. Viral titers were determined by 50% tissue culture infective dose (TCID_50_) assays at the indicated time points postinfection (p.i.). Error bars represent the standard error of the mean values from three replicates. (G) Survival plots for larvae infected with viruses containing wild-type or CRAC-mutated GP64. B. mori instars (day 1 fifth larvae, Liangguang 2 strain, *n* = 60) were injected with 8 × 10^4^ TCID_50_ BVs per larvae, death was recorded at 8-h intervals, and the median survival time was determined by SPSS 22.

In recent years, an increasing number of studies have shown that Bombyx mori nucleopolyhedrovirus (BmNPV) infection depends on cell cholesterol and lipids (1–6), but the mechanism remains largely unknown. Several motifs of membrane proteins have been found to bind cholesterol (7). In baculovirus infection, the cholesterol recognition amino acid consensus (CRAC) domain of GP64 is essential for Autographa californica multiple nucleopolyhedrovirus (AcMNPV) infectivity (8). These CRAC domains were found in BmNPV GP64 (Fig. 1A), and an additional CRAC0 domain was predicted in the signal peptide (SP). SPs are short peptides located in the N terminus of proteins that lead to protein secretion. Typical SPs include the n-region, h-region, and c-region (9), and SPs are removed cotranslationally or posttranslationally in the endoplasmic reticulum. However, we found that the SP of BmNPV GP64 was not cleaved from the mature protein or virion. BmNPV GP64 contains a typical SP, while the n-region is absent in AcMNPV GP64, and the n-region was identified as a minicistron that negatively regulates AcMNPV GP64 translation (10). We found that the n-region blocked SP cleavage of BmNPV GP64. In contrast, the absence of the n-region produced SP-cleaved GP64 (SP^Δn^GP64), which is similar to AcMNPV GP64; however, SP^Δn^GP64 was still assembled into the budding virus (BV). Therefore, the effect of SP retention on GP64 characteristics was investigated via a CRAC bioactivity assay.

## CRAC1 AND CRAC2 ARE REQUIRED FOR BmNPV INFECTION, WHICH IS DETERMINED BY UNCLEAVED SP OF GP64

We generated a series of CRAC mutants based on wild-type *gp64* and SP-cleaved *gp64* (*SP^Δn^gp64*) with point mutant PCR primers (Table 1). The key cholesterol-interacting residue, tyrosine, was replaced with alanine. These mutants were transposed into *gp64*-null bacmids constructed as described in our previous study (Fig. 1B); then, these generated bacmids were transfected into BmN cells, and their activity was measured. As expected, we found that SP retention was key to the unique dependence of GP64 on CRAC domains. We found that GP64 and SP^Δn^GP64 reestablished virus infectivity; however, GP64^Y269A^ and GP64^Y327A^ did not enable *gp64*-null virus infectivity (Fig. 1C), indicating that CRAC1 and CRAC2 are required for GP64 function. In contrast, when SP was cleaved, SP^Δn^GP64^Y269A^, but not SP^Δn^GP64^Y327A^, rescued *gp64*-null virus infectivity (Fig. 1C), which indicated that only CRAC2 is required for SP-cleaved GP64 to exert its effect, corresponding to that of AcMNPV GP64 (8). Surprisingly, the double mutant of CRAC1 and CRAC2 revealed that GP64^Y269&327A^, but not SP^Δn^GP64^Y269&327A^, reestablished infectivity (Fig. 1C), which implied that SP retention alters GP64 folding or structure. Mutations in CRAC0 or CRAC3 in the transmembrane domain did not abolish GP64 or SP^Δn^GP64 function. Western blotting of the GP64-mutated viruses indicated that the CRAC mutants GP64 and SP^Δn^GP64 assembled into BVs (SP^Δn^GP64 was assembled into BVs in the endosomal system [Bifang Hao, Lin Liu, Na Liu, Wenbin Nan, Fengxiu Fan, Jinshan Huang, unpublished data]). There was a significant molecular weight difference in GP64 with cleaved or retained SP (Fig. 1D). Trimerization of the mutants was also determined by nonreducing or reducing gel analysis (Fig. 1E). These data indicated that CRAC mutation did not affect SP cleavage or GP64 trimerization.

**TABLE 1:** Primers used in this study

Name	Sequence (5′ to 3′)
Progp64-F	CGCGAATTCGACAGATATTTAAATAAACCAAAC
Progp64-R	GCGTCTAGATTAATATTGTCTACTATTACGGTT
Y8AF	ATGCTACTAGTAAATCAGTCAGCCCAAGGCTTCG
Y8AR	CGAAGCCTTGGGCTGACTGATTTACTAGTAGCAT
Y269AF	GATCGACAATGATATAGCCGATCTTTCTAAAAACA
Y269AR	TGTTTTTAGAAAGATCGGCTATATCATTGTCGATC
Y327AF	AAGAGGAGCTGATGGCGGAAAACGATTTGCTGAA
Y327AR	TTCAGCAAATCGTTTTCCGCCATCAGCTCCTCTT
Y518AR	GCCCTGCAGTTAATATTGTCTACTATTACGGTTTCTAACCATACAGGCCAAAAATAAAATTACAATAAATAC
L325AF	CATATTCAAGAGGAGGCGATGTACGAAAACGAT
L325AR	ATCGTTTTCGTACATCGCCTCCTCTTGAATATG
L333AF	GAAAACGATTTGCTGGCAATGAACATTGAGCTG
L333AR	CAGCTCAATGTTCATTGCCAGCAAATCGTTTTC

## GP64 SP RETENTION ENDOWS BmNPV WITH HIGH INFECTIVITY

We compared the dynamic production of BVs of the recombinant viruses, and both had typical one-step growth curves; however, the viruses containing uncleaved SP GP64 showed higher BV production than did those with cleaved SP GP64 (Fig. 1F). Two-way variance analysis revealed a significant difference between viruses, and time was determined by SPSS Statistics 22 analysis (*F* = 4.481, *P < *0.001). A repeated-measures analysis of variance was also performed, and the data from five time points followed Mauchly’s test of sphericity (*P = *0.883). These viruses had different proliferation rates (*F* = 4.914, *P < *0.001). Moreover, the larva-killing time for these viruses was determined by intrahemocoelic inoculation with BVs, and the median survival times were determined with SPSS 22. As shown in Figure 1G and Table 2, in addition to the CRAC3 mutant GP64, which showed a significantly prolonged 50% survival time (ST_50_) of 168 ± 1.352 h, BmBac^Δgp64^-gp64 and its mutants exhibited ST_50_ values of 128 to 136 h. However, when SP was cleaved, BmBac^Δgp64^-SP^Δn^gp64 and its mutants exhibited significantly greater ST_50_ values of 152 to 160 h. Significant differences between viruses are reported in Table 3. These results indicated that SP retention in GP64 endowed BmNPV with high virulence and CRAC0 and CRAC3 did not abolish virus infectivity; however, they did affect virus killing. In particular, GP64^Y518A^ significantly prolonged the killing time, showing lower BV productivity than viruses with wild-type GP64, which indicated that CRAC3 mutation in the transmembrane domain affected GP64 activity. This may be caused by the decrease in protein membrane targeting that results from CRAC mutation in the transmembrane domain (11).

**TABLE 2:** ST_50_ values for recombinant viruses^*a*^

Virus	ST_50_ (h)		
	Median	SE	95% CI
BmBac^Δgp64^-gp64	128.000	1.276	125.500–130.500
BmBac^Δgp64^-gp64^Y8A^	136.000	1.265	133.521–138.479
BmBac^Δgp64^-gp64^Y518A^	168.000	1.352	165.350–170.650
BmBac^Δgp64^-gp64^Y269&327A^	136.000	1.320	133.413–138.587
BmBac^Δgp64^-SP^Δn^gp64	160.000	1.661	156.745–163.255
BmBac^Δgp64^-SP^Δn^gp64^Y269A^	160.000	1.675	156.716–163.284
BmBac^Δgp64^-SP^Δn^gp64^Y518A^	152.000	1.352	149.350–154.650

a B. mori instars (day 1 fifth-instar larvae, Liangguang 2 strain, *n* = 60) were injected with a TCID_50_ of 8 × 10^4^ BVs per larvae. Deaths were recorded at 8-h intervals, and the median survival time was determined by SPSS 22.

**TABLE 3:** Pairwise comparisons of the survival times for the recombinant viruses

Virus	Data for comparison with:											
	BmBac^Δgp64^-gp64^Y518A^		BmBac^Δgp64^-gp64		BmBac^Δgp64^-SP^Δn^gp64		BmBac^Δgp64^-SP^Δn^gp64^Y269A^		BmBac^Δgp64^-SP^Δn^gp64^Y518A^		BmBac^Δgp64^-gp64^Y8A^	
	χ^2^	*P*	χ^2^	*P*	χ^2^	*P*	χ^2^	*P*	χ^2^	*P*	χ^2^	*P*
BmBac^Δgp64^-gp64^Y518A^			639.781	0.000	0.616	0.433	0.615	0.433	177.763	0.000	451.942	0.000
BmBac^Δgp64^-gp64	639.781	0.000			475.169	0.000	470.431	0.000	257.569	0.000	54.267	0.000
BmBac^Δgp64^-SP^Δn^gp64	0.616	0.433	475.169	0.000			3.919	0.048	121.273	0.000	308.656	0.000
BmBac^Δgp64^-SP^Δn^gp64^Y269A^	0.615	0.433	470.431	0.000	3.919	0.048			120.490	0.000	306.085	0.000
BmBac^Δgp64^-SP^Δn^gp64^Y518A^	177.763	0.000	257.569	0.000	121.273	0.000	120.490	0.000			120.095	0.000
BmBac^Δgp64^-gp64^Y8A^	451.942	0.000	54.267	0.000	308.656	0.000	306.085	0.000	120.095	0.000		

## GP64 PM LOCALIZATION IS NOT REQUIRED FOR BV PRODUCTION

**FIG 2:**
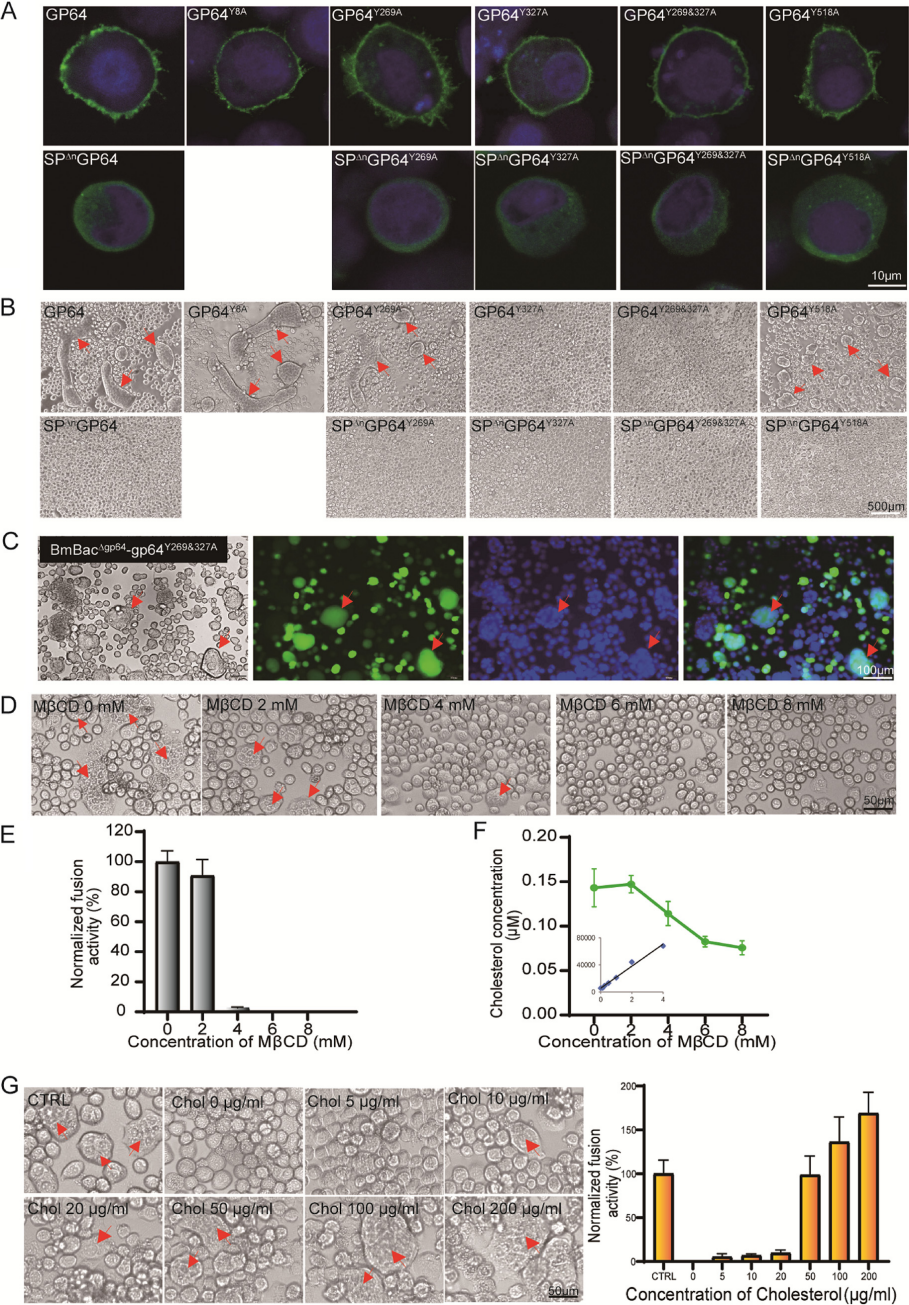
Fusion analysis of CRAC mutants and cholesterol-mediated cell-cell fusion assay. (A) Immunofluorescence analysis of CRAC mutants. BmN cells in the confocal dish were transfected with 2 μg of plasmid harboring the mutants and fixed at 72 h p.t. Immunofluorescence was detected with a GP64 antibody and a fluorescein isothiocyanate (FITC)-labeled secondary antibody, and the nuclei were stained with Hoechst 33258 stain. (B) Cell-cell fusion assay of GP64 and CRAC mutants. BmN cells in a 24-well plate were transfected with 1 μg of plasmid DNA and then incubated at low pH for 5 min to trigger fusion at 72 h p.t.; arrowheads show syncytia. (C) Fusion assay of BmN cells infected with BmBac^Δgp64^-gp64^Y269&327A^. BmN cells were infected with BmBac^Δgp64^-gp64^Y269&327A^ at an MOI of 5, and then cell-cell fusion was induced in low-pH medium 48 h p.i. The images show the light-field, fluorescence, Hoechst 33258-stained, and overlay views. Arrowheads show the syncytia. (D) Fusion assay after cholesterol depletion. BmN cells in a 24-well plate were transfected with 1 μg of wild-type GP64 plasmid DNA, and at 72 h p.t. the cells were incubated with MβCD for 30 min. The chemicals were removed, and fusion was induced. Images show enlarged partial fields. Arrowheads show the syncytia. (E) Efficiency of the inhibitory effect on fusion activity of cholesterol depletion. Fusion efficiency was determined by measuring the percentages of cells found in syncytia. For each treatment, 10 fields were analyzed, and the number of nuclei in the syncytia was divided by the total number of nuclei in the field. Percentages were normalized to parallel syncytium formation data obtained for untreated GP64 (100%). The means and standard deviations are shown in the histogram. (F) Total cholesterol concentration of BmN cells treated with MβCD. BmN cells were incubated with the indicated concentration of MβCD for 30 min, and the total cholesterol level was determined with an Amplex Red cholesterol assay kit (Thermo Fisher Scientific). (G) Cholesterol replenishment rescued fusion in the assay. BmN cells in a 24-well plate were transfected with 1 μg of GP64 plasmid DNA, and at 72 h p.t. the cells were incubated with 8 mM MβCD for 30 min. The cells were washed, and water-soluble cholesterol was added to the medium and incubated for 30 min. The medium was removed, syncytium formation was induced with low-pH medium, and the fusion efficiency was determined as described above. Arrowheads show the syncytia.

To explore why CRAC mutations abolished viral infectivity, we first confirmed that all of the mutants could be expressed and secreted in BmN cells. GP64 and other mutated genes were inserted into the transient expression vector pIZ/V5 (Thermo Fisher Scientific, USA), the generated vectors were transfected into BmN cells, and the expression of the mutants was detected by an immunofluorescence assay. The results showed that GP64 and its mutants were expressed and transported to the plasma membrane (PM) but SP^Δn^GP64 and its mutants were not transported to the PM (Fig. 2A). However, non-PM-localized SP^Δn^GP64 and SP^Δn^GP64^Y269A^ were assembled into BVs, which was inconsistent with BVs acquiring viral membrane proteins and membrane structure from the PM in the budding process (12). This implied that BmNPV has another envelopment mechanism. The DNA virus budding mechanism is more complicated than that of RNA viruses because nucleocapsids are transported from the nucleus (13).

## CRAC2 IS REQUIRED FOR CELL-CELL FUSION MEDIATED BY GP64

Next, we determined the fusion activity of CRAC mutants. GP64 and its mutants were transiently expressed and triggered by low-pH medium at 72 h posttransfection (p.t.), and GP64^Y8A^ and GP64^Y269A^ showed better fusion activity than wild-type GP64; however, the GP64^Y327A^ and GP64^Y269&327A^ mutants failed to promote cell-cell fusion (Fig. 2B), which indicated that CRAC2 is required for cell-cell fusion. Furthermore, when the SP of GP64 was cleaved, SP^Δn^GP64 and its mutants showed no PM localization (Fig. 2A), and no fusion was detected via cell-cell fusion assays (Fig. 2B). Therefore, the fusion activity of SP^Δn^GP64^Y327A^ and SP^Δn^GP64^Y269&327A^ could not be evaluated, underscoring the higher infectivity of the SP^Δn^GP64^Y269A^ recombinant bacmids, and no amplification was found for either SP^Δn^GP64^Y327A^ or SP^Δn^GP64^Y269&327A^ recombinant bacmids. We suggest that CRAC2 is required for SP^Δn^GP64 fusion, with activity corresponding to that of AcMNPV GP64 (8). Although no cell-cell fusion of the GP64^Y269&327A^ mutant was observed in the transient expression assay, fusion of BmN cells infected with the recombinant virus BmBac^Δgp64^-gp64^Y269&327A^ was observed (Fig. 2C), which suggested that other viral factors or host endosomal factors participate in cell-cell fusion, because the viral membrane fuses with the host membrane in the endosome when viruses are endocytosed.

## TARGET MEMBRANE CHOLESTEROL LEVELS ARE CORRELATED WITH CELL-CELL FUSION MEDIATED BY GP64

Cholesterol alters the physical properties of the cell PM in favor of membrane fusion in many enveloped virus infections (14). Since CRAC2 was shown to mediate cell-cell fusion, the role of host membrane cholesterol in cell-cell fusion was investigated. A chemical that interacts with cholesterol was applied in this study; methyl-β-cyclodextrin (MβCD) (Sigma-Aldrich, USA) depletion removed cholesterol from the cell membrane (15). GP64 was transiently expressed in BmN cells. Then, the cells were incubated with the indicated concentrations of drugs, and fusion activity was measured. The results indicated that MβCD application efficiently inhibited cell-cell fusion (Fig. 2D), and the inhibitory effect was enhanced with increasing drug concentration (Fig. 2E). Furthermore, the total cholesterol level in the MβCD-treated cells was determined, and we found that fusion inhibition induced by MβCD was highly correlated with decreasing cholesterol concentration (*r* = 0.900) (Fig. 2F). These results indicated that the cell-cell fusion efficiency mediated by GP64 is correlated with cholesterol in the membrane.

## CHOLESTEROL REPLENISHMENT RESCUED FAILED CELL-CELL FUSION

To further clarify the cholesterol-mediated membrane fusion, GP64-transfected cells were pretreated with 8 mM MβCD. Water-soluble cholesterol was then replenished in the medium, and cell-cell fusion was induced. As expected, cholesterol replenishment successfully rescued fusion failure (Fig. 2G), the fusion efficiency increased with increasing cholesterol concentration, and high-concentration cholesterol replenishment produced fusion activity greater than that observed with wild-type GP64. However, 25-hydroxycholesterol (25-HC) (MedChemExpress, USA) and dehydroergosterol (DHE) (Sigma-Aldrich), analogs of cholesterol, did not rescue fusion failure that had been induced by MβCD depletion (data not shown).

In summary, we suggested that BmNPV GP64, similar to the E1 glycoprotein of Semliki Forest virus (16), is a cholesterol-binding membrane fusion protein. The CRAC domain of GP64 plays an important role in BmNPV infection. CRAC1 is required for viral infection, and we already know the mechanism of its action. CRAC2 mediates cell-cell fusion by interacting with free cholesterol in the target cell membrane, and CRAC0 and CRAC3 are not required but are associated with BmNPV virulence. In contrast to the CRAC2 requirement for AcMNPV infection (8), BmNPV infection requires both CRAC1 and CRAC2, and this difference is a result of SP retention in GP64. Although no BmNPV GP64 prefusion structure is available for GP64 remodeling, other protein structures provide clues. AcMNPV GP64 forms an intermolecular disulfide bridge between Cys24 and Cys372 in the triple-stranded central helix (17); this helical structure is superimposed on rigid blocks composed of the GP64 N terminus in the postfusion structure (18), whereas uncleaved SP of GP64 may stack on these blocks and thus alter the structure. CRAC2 localizes in the α-helical coiled-coil structure in the GP64 postfusion structure (18), while the helix around the CRAC2 region may be partially unfolded in the prefusion state because the stutter may terminate coiled-coil regions (18). In another class III membrane fusion protein, the central helix of vesicular stomatitis virus G (VSV-G) undergoes major refolding during the transition between the prefusion and postfusion structures (19). Here, we suggest that the prefusion BmNPV GP64 CRAC2 domain may not be a part of the long central helix, and this separated CRAC2 domain may contribute to cholesterol binding; solving the structure of BmNPV GP64 will help us to verify this mechanism.

We did not conduct a cholesterol-binding assay in our study because the BmNPV CRAC domain harbors the same residue sequence as AcMNPV, which has been well studied by microarray peptide *in vitro* analysis (8). Although artificial membrane systems and *in vitro* protein-lipid interactions provide informative evidence, these systems lack receptors and do not have a high relative concentration of cholesterol, which are considerable drawbacks to their use (20). As a key cholesterol-binding motif, GP64 CRAC2 mediates cell-cell fusion by interacting with intrinsic or extrinsic free cholesterol on the membrane; however, the cholesterol analogs 25-HC and DHE did not rescue the fusion failure caused by cholesterol removal, which indicates that CRAC2 of GP64 may not interact with DHE *in vivo*. Moreover, CRAC2 point mutations (GP64^L325A^ and GP64^L333A^) did not abolish BmNPV infection, which is inconsistent with the CRAC2 *in vitro* assay results (8) and may be a specific characteristic of BmNPV GP64. Our results were similar to those of other alphavirus studies; cholesterol, but not cholesterol analogs (21), is critical for membrane fusion (20). Double mutation (GP64^Y269&327A^) produced an infectious virus that also exhibited SP retention; although GP64^Y269&327A^ did not promote the fusion of transfected cells, a profound fusion effect was observed in the virus-infected cells. Coincidentally, the same phenotype was observed in the viruses with BmNPV GP64 fusion loops that had been mutated in our previous study (Bifang Hao, Lin Liu, Na Liu, Wenbin Nan, Fengxiu Fan, Jinshan Huang, unpublished data). We found that macropinocytosis was employed by BmNPV to invade host cells (1, 22), and virions were transported into endolysosomes, in which GP64 interacted with the Niemann-Pick type C1 (NPC1) transporter (5). NPC1 is a cholesterol transporter that interacts with Ebola virus glycoprotein priming by cathepsin L in the endolysosome and mediates virus infection with other host factors (23); thus, cholesterol, NPC1, GP64, or other viral proteins may cooperate to mediate BmNPV entry. These results imply that the membrane fusion mediated by BmNPV GP64 with uncleaved SP is far more sophisticated than we previously thought.
